# Patient and family experiences of lysosomal storage diseases in Canada: A qualitative interview study

**DOI:** 10.1002/jmd2.12403

**Published:** 2023-12-29

**Authors:** Nahya Awada, Martin Holcik

**Affiliations:** ^1^ School of Public Policy and Administration Carleton University Ottawa Canada; ^2^ Department of Health Sciences Carleton University Ottawa Canada

**Keywords:** access, health care, lived experience, lysosomal storage diseases, orphan drugs, patient advocacy, patient engagement, rare diseases, reimbursement, social care

## Abstract

Canadian patients and families affected by rare genetic lysosomal storage diseases (LSDs) suffer from numerous challenges related to disease management, including issues navigating healthcare and social support services, access to orphan drugs, and intensive treatment regimens. These challenges significantly impact people's quality of life, yet they remain obscure and have not been the subject of comprehensive analysis. Thus, we conducted qualitative interviews with Canadian patients and caregivers living with LSDs to advance current understanding of their experiences with rare‐disease (RD) management and health systems navigation to support patient‐focused RD policies and programs and improve the health outcomes of the 2.8 million Canadians affected by RDs. This study employed a qualitative descriptive research design with inductive thematic analysis. The study data were collected using semi‐structured interviews. Thirty Canadian participants were interviewed in person or remotely via video chat to allow for an interactive discussion and the acquisition of rich data related to the insights and perceptions of people with LSDs. Between April and November 2019, 30 participants (16 patients and 14 caregivers) with experiences with nine types of LSDs and living in seven Canadian provinces were interviewed. Five themes were identified using comprehensive thematic analysis. These themes were the complexity of the diagnosis process; navigation of healthcare systems; psychological, social, and financial implications of LSDs; access to social support services; and access to orphan drugs. Our findings reveal that patients' access to appropriate healthcare and social services is subject to significant delays and lacks care coordination. The process of accessing orphan drugs in Canada is extremely complex and convoluted. The study results also illuminate experiences of RD stigma when navigating healthcare and social support systems. Our study offers new insights into the complex nature and extensive needs of Canadians with LSDs that are currently unmet. The management of these complex diseases requires holistic patient care and support beyond having access to orphan drugs. Our findings highlight the importance of bridging existing gaps between health and social care for RD patients. Policymakers should utilize these results when developing the forthcoming national RD strategy.

**Synopsis**

The study delved into the challenges faced by Canadian patients and caregivers affected by rare genetic lysosomal storage diseases (LSDs), shedding light on the intricacies of disease management and healthcare system navigation. Conducted through qualitative interviews with 30 participants across seven Canadian provinces, the research uncovered five key themes: the complexity of the diagnosis process, navigating healthcare systems, psychological, social, and financial impacts of LSDs, access to social support services, and access to orphan drugs. Findings revealed significant delays and fragmented care in accessing healthcare and social services, intricate hurdles in obtaining orphan drugs, and experiences of stigma within healthcare and support systems. These insights emphasize the unmet needs of individuals with rare diseases, stressing the necessity for comprehensive care beyond access to orphan drugs, urging policymakers to bridge gaps in health and social care to better support these patients within the forthcoming national strategy for rare diseases in Canada.

## 1 BACKGROUND

Studies of rare diseases predominantly investigate specific biomedical aspects of these diseases, such as the ways in which they are diagnosed or treated. Many studies have also been conducted to examine the experiences of patients with rare diseases and their families.^1^, ^2^, ^3^, ^4^, ^5^, ^6^ Inherited metabolic diseases (IMD) are sometimes the focus of such studies.^7^, ^8^, ^9^, ^10^ However, the common psychosocial and economic experiences of individuals with lysosomal storage diseases (LSDs) have not been comprehensively studied or analyzed through an academic lens^11^ despite growing recognition that understanding patient and family experiences is paramount to providing adequate patient‐centered care.^12^ Although LSDs are a category of IMD, the diagnosis, disease manifestations, complications, and treatment regimen of LSDs are different from those experienced in other types of IMDs or other rare conditions.

Canada is one of the few developed countries that has not adopted a rare‐disease strategy to promote health outcomes and management of rare diseases nor an orphan drug legislation to facilitate the research and development of and accessibility to orphan drugs.^10^, ^13^, ^14^ In 2019, the Canadian government declared its intention to establish a national strategy for high‐cost drugs for rare diseases. This national strategy aims to provide consistent and efficient access to the drugs needed by Canadians with rare diseases.^15^, ^16^ Although a detailed plan for developing and implementing this strategy has not yet been developed, the federal budget for 2019 included funding of up to 500 CAD million per year over 2 years to develop this national strategy, beginning in 2022–2023.^15^, ^16^ In a Speech from the Throne in September 2020, the government emphasized commitment to a rare‐disease strategy that addresses diagnosis, treatment, and quality of care for Canadians with rare diseases rather than a narrower‐focused orphan drug strategy. Canada is thus uniquely positioned to develop an evidence‐based national rare disease strategy that includes an orphan drug framework. This strategy could heed the lessons of approaches to managing rare diseases and orphan drugs that have been employed by other developed countries. It could also incorporate lessons from Canadian patients' experiences with the healthcare systems and social support services. Understanding the experiences, preferences, and needs of Canadians with complex rare diseases, such as LSDs, is imperative in this respect, as these first‐hand insights could help ascertain which foreign policies and programs are relevant to the needs of Canadian patients and their families. This understanding also helps to identify potential service gaps and highlight factors that influence negative patient health outcomes.^12^, ^17^

This qualitative interview study was designed to gain insights from experiences, needs, and challenges faced by patients with LSDs and their families. The study reinforces existing research into rare diseases in Canada by addressing gaps in the existing knowledge base regarding the experiences of this patient population, including management of these rare conditions, navigation of healthcare and social systems, and access to orphan drugs. Furthermore, the type of patient input collected through our study would serve as reliable baseline data, which could be subsequently used for comparative analyses and evaluation after implementation of the new RD strategy. This would ensure new programs and policies, particularly the forthcoming national rare‐disease strategy, achieve an appropriate level of performance impact, ensure goals are met, and help devise and implement any necessary changes.

## 2 METHODS

### 2.1 Design, sampling, and recruitment

We conducted qualitative, semi‐structured interviews with 30 participants, after which saturation of data was reached, and we were satisfied that the data collected had addressed all of the emerging themes. In this study, data saturation was defined as the point at which the sample included adequate representation of most types of LSDs for which orphan drugs are available, included residents of most Canadian provinces, and additional interviews merely repeated common themes and did not illuminate any new ideas.

The study employed purposive, non‐probability sampling to identify participants who had lived experiences with LSDs. Eligibility criteria included a residency in one of the 10 Canadian provinces and a confirmed diagnosis of LSD for either the participant themselves or their child. All participants were over 25 years of age, and they all had the intellectual abilities to participate in the study. This ensured legal consent could be obtained and that the sample sufficiently incorporated a diverse range of ages. Individuals who did not speak English and/or who were unable to participate in interactive interviews were excluded from this study, as were the parents of children who had died three or more years before the study was initiated. The study participants all had experiences with the rare disease journey and had experienced several aspects of healthcare delivery in Canada, including access to therapies, healthcare, and social services. Participants' ethnicity, level of education, employment, and marital status were disregarded, and the availability of an orphan drug in Canada was not included in the inclusion criteria, which maximized the diversity of the sample.

Following ethics approval from the Carleton University Research Ethics Board (CUREB; Project# 110105; See Appendix S1), letters were distributed to different Canadian organizations, foundations, and associations concerned with rare diseases and LSDs. These letters asked recipients to share information about this study with their members. Many of these organizations agreed to promote the study and shared relevant information with their members through emails, websites, social media platforms (e.g., Facebook, Twitter), and newsletters. This widespread distribution of information ensured participants from across Canada were aware of the study.

Forty candidates expressed an interest in participating in this study, three of whom were ineligible: one participant could not speak English, and the other two prospective participants were the parents of children who had died 12 and 26 years earlier. A further seven candidates withdrew from the study prior to interviews. Semi‐structured interviews were subsequently conducted with 30 participants (16 patients and 14 parents of children with LSDs). This number was sufficient for reaching data saturation in the preliminary analysis.

Respondents were asked to provide their informed consent (written or verbal) to participate in the study, and the proposed interview questions were provided in advance. Interviews were conducted in person or using a remote video‐chat service (i.e., Skype, FaceTime, WhatsApp). This direct communication facilitated interactive discussions that yielded a deep exploration of each individual's experiences,^18^ including rich, comprehensive data relating to the experiences and perceptions of people living with LSDs. However, two participants asked for the interview to be conducted via teleconference, and these requests were accommodated. Probing techniques were employed during the interviews when it was necessary to encourage participants to elaborate on their personal experiences.

The interview questions were designed to capture information related to participants' experiences with rare diseases, including diagnosis, disease management, and navigation of the healthcare system. The interviewees were also asked about the social, psychological, and financial impact of their rare diseases on their daily lives, and they were asked to describe their access to and interactions with social support and healthcare systems and providers. Finally, the interviews explored participants' access to orphan drugs and engagement in drug reviews and regulatory decision‐making processes, as well as their involvement in other stages of the orphan drug lifecycle (i.e., pre‐clinical trials, clinical research, and post‐marketing studies).

### 2.2 Data collection

The main researcher (N.A.) conducted all the interviews using a semi‐structured interview format (Appendix S2) to guide the discussion of a broad range of relevant patient experiences that had been gleaned from the academic literature. The audio of all interviews was recorded and then transcribed using Temi transcription software. The researcher subsequently verified the interview transcripts by simultaneously listening to each audio recording and reading its corresponding transcript.

### 2.3 Data analysis

This study employed a qualitative descriptive analytical approach. This method is considered appropriate for this study because it provides intricate insights into participants' experiences, which are expressed in their own words and minimizes the need for interpretation.^19^

Preliminary data analysis of the first three interview transcripts resulted in the identification of several general conceptual categories. More specific concepts were then identified and coded using NVivo software (version 12), which were then reviewed by the study team to confirm their trustworthiness, including their credibility, transferability, dependability, and confirmability.^20^

As the data collection and analysis progressed, key themes emerged from the conceptual categories, which were further explored and emphasized in later interviews. The thematic approach used to analyze the data in this study draws from the six phases of thematic analysis outlined by Braun and Clarke.^21^ To further enhance and clarify data contained in the themes, information was broken down into sub‐themes which identified specific aspects of the participants' experiences.^21^

## 3 RESULTS

### 3.1 Participants

The 30 semi‐structured interviews were conducted between April 1 and November 15, 2019. Each interview lasted an average of 70 min. Fourteen (46.7%) participants were the parents of children affected with LSDs, and 16 (53.3%) were adult LSD patients over 25 years of age. Of the 14 parents interviewed, three (21.4%) had more than one child affected with an LSD. Similarly, of the 16 adult patients interviewed, three (18.7%) had at least one sibling affected with the same LSD. Table 1 presents an overview of each participant's number, diagnosis, and relationship to the disease (i.e., status as a patient or parent of a patient). Because the number of Canadians affected with each type of LSD may be less than five patients per province, the participants' province of residence, gender, and age were omitted from the table to protect their identity.

**TABLE 1:** Participant information.

Participants	Patient diagnosis	Participant's status	Patient receiving orphan drug?	Time to access orphan drug following diagnosis	Source of funding
Participant 1: P1	MPS IV	Parent	Yes, on ERT	2–3 years	Government
Participant 2: P2	Pompe	Patient	Yes, on ERT	Less than 1 year	Government
Participant 3: P3	MPS I	Parent	Yes, on ERT	Less than 1 year	Compassionate use
Participant 4: P4	Pompe	Patient	Yes, on ERT	4–5 years	Government
Participant 5: P5	MPS IV	Parent	Yes, on ERT	7–8 years	Compassionate use
Participant 6: P6	Pompe	Patient	Yes, on ERT	Less than 1 year	Government
Participant 7: P7	MPS I	Patient	No	Denied access	Not applicable
Participant 8: P8	Pompe	Patient	Yes, on ERT	9–10 years	Private insurance
Participant 9: P9	MPS II	Parent	Yes, on ERT	Less than 1 year	Compassionate use
Participant 10: P10	Pompe	Patient	Yes, on ERT	Less than 1 year	Clinical trial
Participant 11: P11	Pompe	Patient	Yes, on ERT	26 years	Government
Participant 12: P12	Fabry	Patient	Yes, on ERT	Less than 1 year	CFDI^b^
Participant 13: P13	Pompe	Parent	Yes, on ERT	1–2 years	Private insurance
Participant 14: P14	MPS IV	Patient	Yes, on ERT	14–15 years	Private insurance
Participant 15: P15	Fabry	Patient	Yes, on ERT	28–29 years	Compassionate use then CFDI^b^
Participant 16: P16	MPS I	Parent	Yes, but stopped after BMT	Less than 1 year	Government
Participant 17: P17	MPS III	Parent	No	Not Applicable^a^	Not applicable
Participant 18: P18	Pompe	Patient	Yes, on ERT	Less than 1 year	Private insurance
Participant 19: P19	Pompe	Patient	Yes, on ERT	4–5 years	Government
Participant 20: P20	MPS IV	Parent	Yes, on ERT	Less than 1 year	Government
Participant 21: P21	Fabry	Patient	Yes, on ERT	Less than 1 year	Compassionate use then CFDI^b^
Participant 22: P22	Gaucher	Patient	Yes, but stopped after ERT infusion reaction	Less than 1 year	Government
Participant 23: P23	Alpha Md	Parent	No	Not Applicable^a^	Not applicable
Participant 24: P24	MPS III	Parent	No	Not Applicable^a^	Not applicable
Participant 25: P25	MPS III	Parent	No	Not Applicable^a^	Not applicable
Participant 26: P26	MPS I	Parent	Yes, but stopped after BMT	Less than 1 year	Private insurance
Participant 27: P27	MPS II	Parent	Yes, but stopped when private insurance ended	1–2 years	Private insurance
Participant 28: P28	Pompe	Patient	Yes, on ERT	8–9 years	Government
Participant 29: P29	Fabry	Patient	Yes, ERT then SRT	3–4 years	CFDI^b^
Participant 30: P30	MPS VI	Parent	Yes, on ERT	Less than 1 year	Government

^a^ No orphan drug was available (i.e., not approved for marketing nationally or internationally) at the time of interview.

^b^ The Canadian Fabry Disease Initiative (CFDI) is a national study that started in 2007 where Fabry patients were provided with publicly funded access to either Fabrazyme (agalsidase beta) or Replagal (agalsidase alfa) on a randomized basis.

**FIGURE 1:**
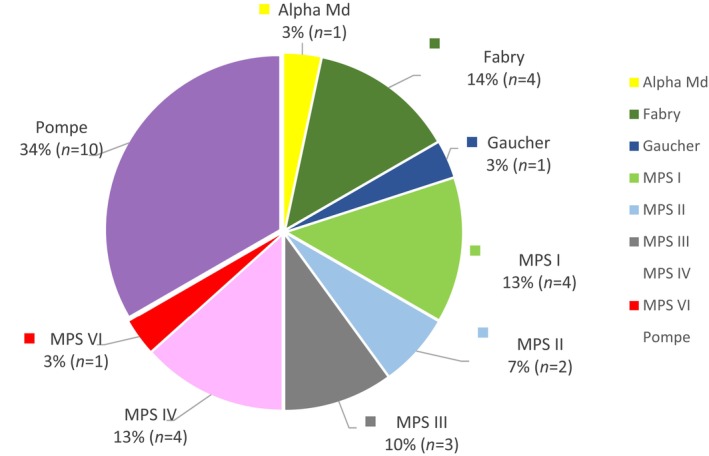
Participants' diagnosis by lysosomal storage disease (LSD) type. The proportion of participants (total number and percentage) diagnosed with each of the nine types of the LSDs included in the study. Mucopolysaccharidosis (MPS) type I (Hurler, Hurler/Scheie Syndrome), MPS type II (Hunter Syndrome), MPS Type III (Sanfilippo), MPS Type IV (Morquio Disease), MPS Type VI (Maroteaux‐Lamy Syndrome), and Alpha‐mannosidosis (Alpha Md) disease.

**FIGURE 2:**
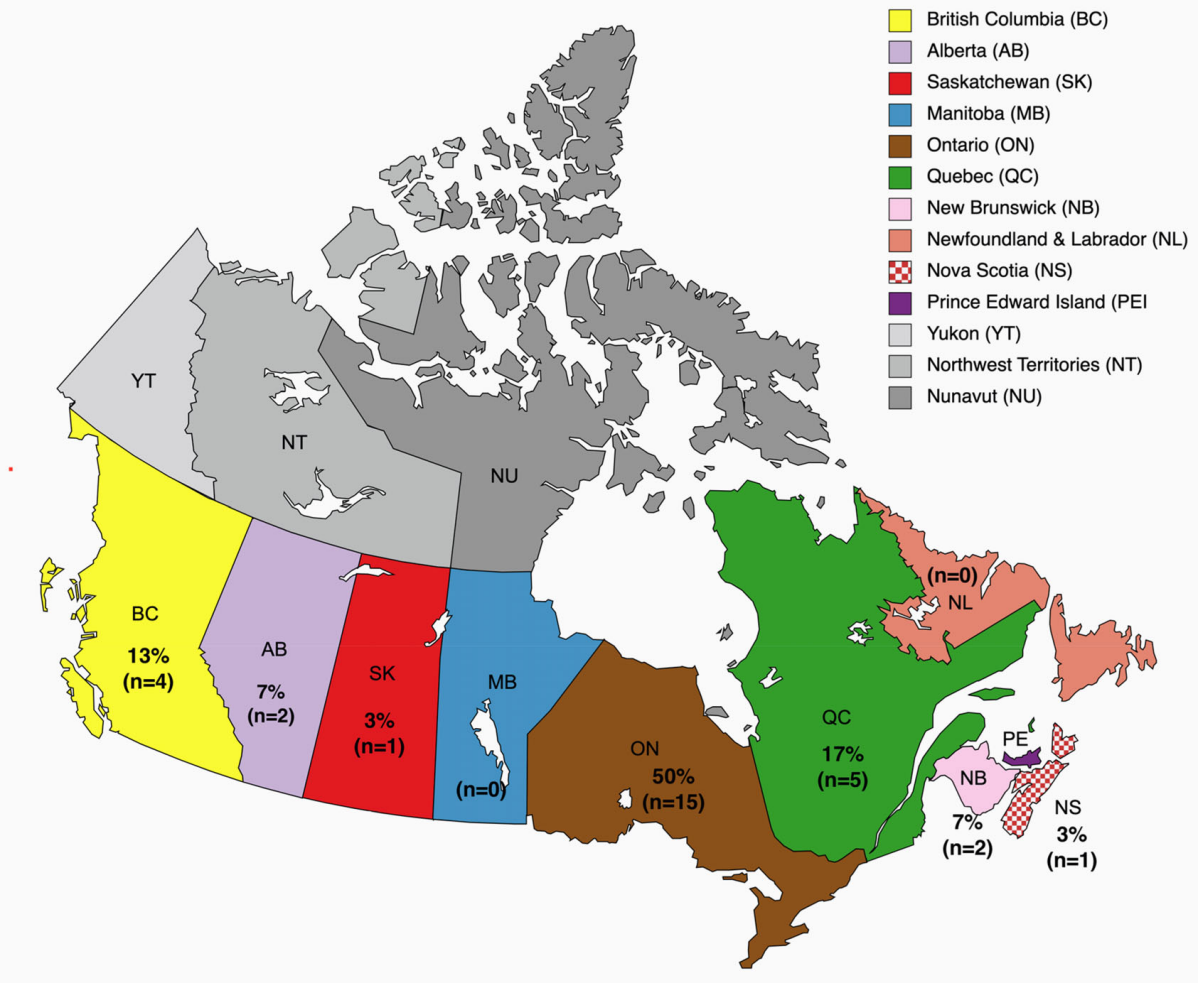
Geographic distribution of research participants by province (percentage & number).

The study included 30 participants affected by nine different types of LSDs, as depicted in Figure 1. These participants were spread across seven different Canadian provinces, as illustrated in Figure 2.

All parents (two fathers and 12 mothers) who participated in this study were the main caregivers of their LSD‐affected children and had thus experienced myriad aspects of the rare‐disease journey in the previous 3 years, including diagnosis, access to therapy, and ongoing interactions with healthcare services and providers. These parents' insights were thus deemed useful insights into rare‐condition management and healthcare systems. The diseases represented by the participants are summarized in Table 2.

**TABLE 2:** Diseases represented by participant status (parent vs. patient).

Diagnosis	Number		
	Patient	Parent	Total
MPS I (Hurler)	1	3	4
MPS II (Hunter)	0	2	2
MPS III (Sanfilippo)	0	3	3
MPS IV (Morquio)	1	3	4
MPS VI (Maroteaux‐Lamy)	0	1	1
Pompe	9	1	10
Fabry	4	0	4
Gaucher	1	0	1
Alpha‐mannosidosis	0	1	1
Total	16	14	30

### 3.2 The experiences of patients with LSDs and their families: participants' perspectives

Although there was heterogeneity within the sample in terms of each participant's type of LSD, age group, province of residence, and the availability and accessibility of corresponding orphan drugs, participants' common perceptions of the challenges and needs have resulted in five overarching themes. The key themes, which were categorized using a comprehensive thematic analysis, include (1) the complexity of the diagnosis process; (2) navigation of healthcare systems; (3) the psychological, social, and financial implications of LSDs; (4) access to social support services; and (5) access to orphan drugs. Sub‐themes were similarly identified based on the conceptual categories in the coded data and are discussed as they appear within the key themes (Table 3).

**TABLE 3:** Themes, subthemes, and key findings.

Themes	Subthemes	Key findings
The complexity of the diagnosis process	Delayed diagnosis	The timeline within which participants received a correct diagnosis ranged from less than 1 year to more than 40 years. Most participants (30%, *n* = 9) received an accurate diagnosis between 1 and 5 years. Every participant in this study received at least one incorrect diagnosis, with some received more than 20 wrong diagnoses before receiving a correct one. Most early diagnosis cases (i.e., diagnosis received in less than 5 years time from the time of onset of symptoms) occurred with early onset LSDs (e.g., MPS disorders), where the diagnosis was triggered by developmental delays, distinctive facial features, and/or physical and growth abnormalities.
	2 Consequences of delayed diagnosis	Delayed diagnosis and misdiagnosis contributed to worsening clinical conditions and outcomes and, at times, had resulted in severe, irreversible, and, sometimes, life‐threatening consequences.
II Navigation of healthcare systems	Access to healthcare services	A range of barriers to accessing the healthcare services needed to manage LSDs were identified. These barriers include medical professionals' limited knowledge about rare diseases, lack of service coverage by patients' respective healthcare systems, specialist health services being unavailable in patients' regions of residence, long waiting times to see specialists and health professionals, and the financial barriers generated by the disease management and manifestations.
	2 Coordination of care	Patients and caregivers spent significant amount of time each day coordinating medical appointments and relaying information between a range of general medical professionals and rare‐disease specialists. The time and effort required for care coordination and rare‐disease disease management impacted participants' quality of life and contributed to their psychosocial and financial burden.
	3 Interaction with the medical community	Negative experiences with the medical community were common. These experiences included insufficient guidance for patients and their families following diagnosis, the medical community's lack of knowledge about rare diseases, the lack of patient engagement in health decisions, difficulties convincing physicians that patient's symptoms were real, and specialists' reluctance to submit an orphan drug application on behalf of patients. The negative experiences with the medical community contributed to participants' mental and financial distress and, at times, led to health deterioration.
	4 Access to disease‐ and system‐ related information	Participants received little to no guidance and information upon diagnosis about their condition, health system navigation, available social support services and resources, patient organizations, and research opportunities. The lack of informational support resulted in severe emotional, social, and financial hardship, particularly in the initial years following diagnosis.
III. The psychological, social, and financial implications of LSDs	The psychological implications of LSDs	Anxiety, stress, emotional exhaustion, and depression were all identified within this study's patient population Stress and anxiety levels were exacerbated by the complex diagnostic process, difficult access to orphan drugs, lack of information and guidance upon diagnosis, uncertainty surrounding prognosis, and feelings of alienation within the healthcare system (i.e., interpersonal and structural stigma). Considerable, various feelings of loss, which were the result of LSDs' manifestations and management, were reported. The feelings of loss included loss of friends, freedom, autonomy, and employment.
	2 The social implications of LSDs	Social isolation is a major social challenge experienced throughout the LSD journey. This isolation included social distancing and drawbacks of friends and family members following diagnosis. Disease manifestations (e.g., chronic pain and fatigue) and management (e.g., frequent medical appointments, ERT weekly infusions) also impacted on patients' social lives and contributed to social isolation. Care coordination responsibilities held by patients and caregivers is a major barrier to social life. The social impact of LSDs was higher for the parents of children whose LSDs involve mental or physical disabilities.
	3 The financial implications of LSDs	LSDs generate serious economic repercussions for patients and their families in numerous ways, many of which were not overt. These financial repercussions stem from: (1) inability to maintain paid employment and reduced patient or family income; (2) travel costs for medical purposes; (3) disability‐related expenses (i.e., costs associated with adapting houses and/or vehicles, caring of children with disabilities); and (4) out‐of‐pocket medical expenses (i.e., emergency kits, uncovered health services). The emotional burden placed on patients with LSDs and their families was augmented by the significant financial burden.
IV Access to social‐support services	Access to psychological support	Lack of professional psychological support throughout patient's journey with the disease. The role of patient organizations and groups was acknowledged in this respect. These entities' activities and platforms, (e.g., support groups, conferences) provided patients the opportunity to speak to people with similar experiences, which often reduced their feelings of isolation and abnormality. These activities also facilitated information exchange between people affected by the same condition, which helped them learn about their conditions, available resources, navigate health and social systems, and manage their specific diseases.
	2 Access to tangible support	Tangible support, which was reported as one of the critical elements of disease management, included four specific needs: (1) supported employment; (2) travel expenses; (3) respite services; and (4) disability support programs and services (i.e., assistance for the families of children with disabilities, assistance for adult patients with disabilities, respite services, and adapted housing and vehicles). Participants received some form of financial assistance from the government, but this varied significantly between provinces. The received support was insufficient.
V Access to orphan drugs in Canada	The drug application process	Study participants across all provinces, including the ones that established specific programs to facilitate access to orphan drugs such as Ontario, Alberta, New Brunswick, and British Columbia, shared similar challenges and experiences with regards to accessing orphan drugs. The treating physician is responsible for submitting orphan drug application following diagnosis, follow‐up on reimbursement decision with relevant regulatory bodies, and drug renewals applications. No formal appeal process exists to respond to negative decisions concerning orphan drugs' reimbursement. The appeal of negative decisions was done with the help of the treating physicians and/or patient organizations. The media was also used to pressure decision makers to reconsider their decisions.
	2 Challenges and barriers to accessing orphan drugs	The barriers to accessing orphan drugs, which were encountered in all provinces, include: (1) overly complex, vague, and confusing drug application process; (2) unknown and/or convoluted eligibility criteria for drug reimbursement; (3) patients' sickness and inability to self‐advocate; (4) complex administrative procedures for ERT infusion administration and drug reimbursement strategy (particularly for privately funded orphan drugs); and (5) physicians' reluctance to submit drug applications.
	3 Patient engagement in the orphan drug lifecycle	Participants were keenly interested in engagement in all stages of the orphan drug lifecycle. Opportunities for patient engagement in activities related to regulatory review for orphan drugs were scarce and informal. There is little to no engagement in the decision‐making processes concerning drug reimbursement, which was conducted on a case‐by‐case basis. Patient engagement in research activities was limited to participation in clinical trials, joining registries, surveys' completion, and providing samples to biobanks.

#### 3.2.1 Theme 1. The complexity of the diagnosis process

**FIGURE 3:**
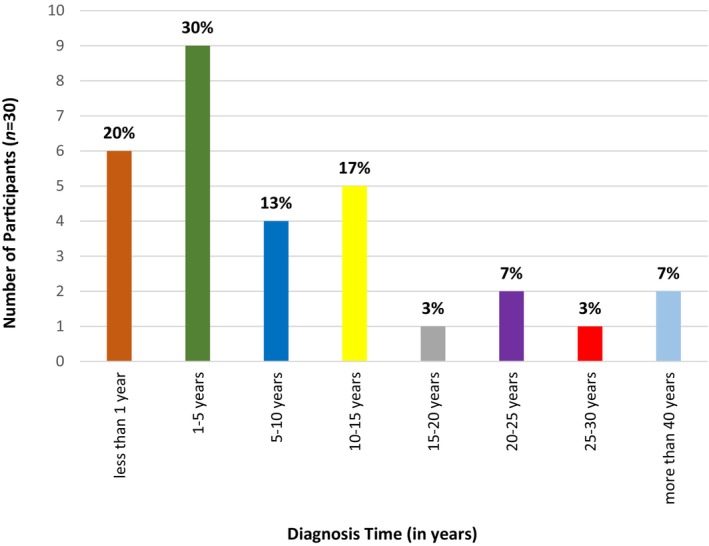
Time to obtain an accurate diagnosis. This figure illustrates the time (in years) participants waited to receive an accurate diagnosis.

Obtaining an accurate diagnosis can be a long journey for many patients with LSDs. This complex process, commonly known as the “diagnostic odyssey,” refers to the time between the onset of symptoms and the procurement of a definitive diagnosis. The participants in this study related distinct experiences of their diseases being diagnosed, depending on the patient's age, the severity of their symptoms, and the type of LSD. The timeline within which participants received a correct diagnosis ranged from less than 1 year (20%, *n* = 6) to more than 40 years (7%, *n* = 2), with most participants receiving a diagnosis between 1 and 5 years (30%, *n* = 9) (Figure 3).

##### Subtheme 1: Delayed diagnosis

Although all LSDs are progressive, each disorder varies in terms of its rate of progression, severity of symptoms, and affected organs. The variable phenotypes and genotypes of LSDs, along with the rarity of these conditions, contribute to delays in their accurate diagnoses. Other contributing factors to delayed diagnosis include: (1) the age of disease onset, (2) absence of familial history of LSD, (3) lack of knowledge within the medical community about rare diseases, and (4) laboratory testing‐associated errors.

Every participant in this study received at least one incorrect diagnosis, and some received more than 20 misdiagnoses before their rare disease was correctly identified (Box 1, [1a]). Notably, most early diagnosis cases occurred during childhood phases when developmental delays, distinctive facial features, and/or physical and growth abnormalities triggered the diagnostic process (Box 1, [1b]).

Most participants indicated that they had consulted more than five healthcare professionals before ultimately receiving a correct diagnosis. Patients with more complex and unpredictable symptoms reported a greater number of medical consultations and more extensive diagnostic testing.

Remarkably, this study also revealed how the odyssey through delayed and misdiagnosis has changed very little over the last few decades despite improvements to diagnostic technologies and tools. For instance, a participant who, for over 43 years, experienced many symptoms before receiving an accurate diagnosis of Fabry disease in 2001 (Box 1, [1c]) shared similar experiences and challenges as another participant who waited 20 years before being diagnosed with Gaucher in 2015 (Box 1, [1d]).

##### Subtheme 2: Consequences of delayed diagnosis

According to participants, obtaining an accurate diagnosis is life‐changing. It helps patients and parents better understand their condition, associated symptoms, and prognosis. An accurate diagnosis also halts unnecessary diagnostic testing and contributes to the provision of unnecessary diagnostic testing and contributes to providing focused medical care and appropriate social support. Patients perceived delayed diagnosis as a major problem contributing to worsening clinical conditions and outcomes (Box 1, [2a]). Several participants indicated that the delayed diagnosis resulted in severe, irreversible, and sometimes life‐threatening consequences (Box 1, [2b]).

#### 3.2.2 Theme 2. Navigation of healthcare systems

The multidisciplinary nature of managing LSDs necessitates patient access to a wide range of healthcare providers and specialists, which requires a holistic approach and coordinated care (Box 2, [1a]). This theme encompasses four subthemes: access to healthcare services, the level of coordinated care they received, their interactions with the medical community, and participants' experiences of accessing disease‐ and system‐related information.

##### Subtheme 1: Access to healthcare services

Interviews conducted for this study reveal that patients utilized an extensive range of specialist medical services (e.g., pediatricians, geneticists, ophthalmologists, dentists, neurologists, surgeons) and healthcare‐support services (e.g., speech therapy, physiotherapy, occupational therapy) (Box 2, [1b]). Throughout their rare‐disease journeys, patients with LSDs often required both invasive and non‐invasive procedures (e.g., Port‐a‐Cath insertion, heart surgery, orthopedic surgery, dental repair). Some participants recounted numerous emergency room visits, including hospital admission to treat symptoms before their condition was diagnosed. Furthermore, patients with LSDs periodically visited a broad range of healthcare centers to undergo various tests (e.g., 6‐min walk test, 3‐min climbing stairs test, pulmonary function test, ophthalmic exam, kidney function test). Such tests were required both to monitor the patient's ongoing disease management and to continue receiving funding for an orphan drug, which depended on validating the patient's continued eligibility. Moreover, LSD patients receiving enzyme replacement therapy (ERT) infusions needed to attend appointments to receive lifelong weekly or bi‐weekly intravenous infusions from trained healthcare providers.

The participants encountered a range of barriers to accessing the healthcare services needed to manage their diseases, including medical professionals' limited knowledge about rare diseases (Box 2, [1c]), lack of service coverage by patients' respective healthcare systems (Box 2, [1d]), specialist health services being unavailable in their regions of residence (Box 2, [1e]), long waiting times to see specialists and health professionals (Box 2, [1f]), and the financial barriers generated by extensive travel costs, parents' arrangements for siblings' care, and absences from work to attend appointments (Box 2, [1 g]).

##### Subtheme 2: Coordination of care

Since LSDs are chronic, progressive, and affect multiple systems of the body, people living with these diseases require ongoing multidisciplinary medical management throughout their lives. It is therefore essential that these varying healthcare providers communicate effectively and coordinate the patient's care efficiently.

In this study, many participants described themselves as full‐time care coordinators because a significant amount of each day is spent coordinating medical appointments and relaying information between a range of general medical professionals and rare‐disease specialists. This was particularly true of caregivers who undertook these responsibilities to ensure their children received safe and adequate care. These parents emphasized that the lack of coordinated care could result in significant safety issues and compromised patient care (Box 2, [2a]).

Most participants affirmed that the time and effort required to coordinate and manage a rare disease impacted their quality of life and contributed to their psychosocial burden (Box 2, [2b]). Participants also asserted that adequately organized and coordinated patient care significantly lessens the costs involved in managing a rare condition (e.g., travel costs incurred by multiple appointments), aids the ability to work, and eases feelings of insecurity surrounding their own or their children's safety and wellbeing.

##### Subtheme 3: Interactions with the medical community

Physicians play a vital role in promoting patient access to care and treatment. This is particularly true with regards to orphan drugs, as applications for such drugs can only be initiated by a medical specialist in Canada. Participants shared varying experiences of their interactions with the general medical community and specialized treating physicians. These experiences ranged from significantly positive to extremely negative.

The accounts of positive experiences included instances of physicians supporting patient's access to orphan drugs and actively advocating for them (i.e., appeal responses to negative reimbursement decisions). The negative experiences, on the other hand, involved ineffective communication with physicians, the dearth of guidance and information offered to patients and their families upon diagnosis, the medical community's lack of knowledge about rare diseases, and the lack of patient engagement in health decisions. Indeed, some patients explained their difficulties when trying to convince doctors that their symptoms were real. Meanwhile, other participants reported being accused of wasting doctors' time or being misdiagnosed with psychological conditions, such as depression (Box 2, [3a]). Some participants pleaded with their treating physician in the hopes of convincing them to submit an orphan drug application on their behalf. For many participants in this study, these negative experiences have contributed to their psychological and financial distress. Such experiences have also made participants feel less respect for and trust in healthcare systems and providers (Box 2, [3b]).

Furthermore, the lack of patient engagement in health decisions, including a sense of medical paternalism, was broached by many participants as a source of significant distress. Participants felt excluded from decisions about their care. They encountered difficulties when asking their treating physicians to initiate treatment, include them in clinical trials, and submit orphan drug applications on their behalf. Some parents felt conflicted about their role as advocates for their children's care, noting that their interactions with physicians were necessarily cautious. Parents were worried that being perceived as bothersome might influence the physician's recommendations for their child's services, treatment, and care (Box 2, [3c]).

##### Subtheme 4: Access to disease‐ and system‐related information

In this study, informational support is defined as patients' and their families' awareness of and knowledge about the patient's rare disease, as well as about related healthcare systems, social services, resources, and activities that affect their disease management (e.g., clinical trials, registries). For study participants, informational support was described as critically important. However, this study reveals several challenges that impede access to the information people with rare diseases require to manage their diseases. Participants emphasized the need for more information upon diagnosis, particularly with regards to symptom management, treatment options, and disease progression. This information, they noted, would significantly enhance their ability to make informed decisions about care plans (Box 2, [4a]). These patients and their families also asserted that diagnosis was not accompanied by information about available social support, resources, patient organizations, and research opportunities, and they reportedly received poor guidance concerning effective and efficient navigation of health and social‐support systems. For patients and their families, this lack of information resulted in severe emotional, social, and financial hardship, particularly in the initial years following diagnosis (Box 2, [4b]). Subsequently, these participants expressed the need for increased information about diseases, relevant health and social services, and patient organizations, which should be routinely offered to families following diagnosis.

#### 3.2.3 Theme 3. The social, psychological, and financial implications of LSDs

Although LSDs involve a wide variety of symptoms and prognoses, these conditions share several common aspects. For example, LSDs often involve multi‐system dysfunction and delayed diagnosis, they frequently require multidisciplinary and complex care, treatment is often expensive, and the disease is often incurable. In addition, many types of LSDs are also associated with motor, sensory, and/or intellectual impairment, and they impose significant social, psychological, and financial burdens on patients and their families.

##### Subtheme 1: The psychological implications of LSDs

Many participants described the ways in which living with a chronic, progressive, and degenerative condition, such as an LSD, detrimentally affected their psychological wellbeing. Anxiety, stress, emotional exhaustion, and depression were all identified within this study's patient population. Although these psychological states can be experienced with any chronic condition, rare diseases can amplify the mental impact on patients and their families. Study participants outlined numerous factors that resulted in increased levels of stress, but the diagnosis odyssey (Box 3, [1a]) and difficult access to orphan drugs (when available) (Box 3, [1b]) were often emphasized as among the most significant. Stress and anxiety levels were frequently exacerbated by the lack of information available upon diagnosis, uncertainty surrounding their prognosis, and feelings of alienation within the healthcare system.

Participants' psychological distress also resulted from the challenges involved in the day‐to‐day management of their diseases (Box 3, [1c]), as well as issues relating to acquiring and maintaining employment as these illnesses often required frequent absences from work. Participants whose orphan drugs were funded by private insurance that was dependent on their employment often encountered additional pressure and stress from employers. These participants felt that they had no alternative to enduring their employer's pressure so that they could continue receiving both an income and funding for their orphan drug (Box 3, [1d]).

Moreover, participants in this study articulated various feelings of loss that directly related to living with an LSD. These considerable, continuous losses, which were the result of the disease manifestations and management, include losing friends, freedom, autonomy, and employment. This often resulted in participants exhausting their psychosocial and financial resources.

##### Subtheme 2: The social implications of LSDs

Participants reported social isolation as a major social challenge experienced during their rare‐disease journeys. Analysis of participants' responses identified several causes of this social isolation, including the social distancing and drawbacks of friends and family members following a patient's diagnosis and the effects of disease manifestations on patients' social lives (Box 3, [2a]). This was true both for patients with physically visible symptoms (e.g., coarse facial expressions, a pigeon chest, short stature, the use of a wheelchair) (Box 3, [2b]) or invisible symptoms (e.g., chronic fatigue, pain) (Box 3, [2c]). The aspects of disease management also hindered patients' social inclusion and interaction. For instance, the intensity of the treatment regimen (i.e., lifelong weekly/ biweekly ERT infusion) and the frequent medical appointments led to a lack of time and energy to socialize and, subsequently, significantly impacted patients' and their families' daily activities and quality of life (Box 3, [2d]).

It is also important to note that LSDs dramatically impact the social lives of those who care for patients affected by these diseases. In this study, caregivers often reported having to quit their jobs to care for their children. They described their caregiving responsibilities (i.e., coordination of medical appointments, provision of care at home, and communication with educational institutions to accommodate their children's needs) as all‐consuming and a barrier to social life (Box 3, [2e]). The social impact was higher for the parents of children whose LSDs involve mental or physical disabilities.

##### Subtheme 3: The financial implications of LSDs

Study participants outlined the serious economic repercussions of rare diseases, with patients and their families often incurring numerous costs, many of which were not overt.

Most patients in this study noted that dealing with rare diseases had affected their ability to maintain paid employment. These participants reported that their income had been reduced because disease manifestations and management significantly impeded their professional activities, including compromising productivity and the ability to work standard hours (Box 3, [3a]). They further emphasized that long‐term employment plans were hindered by the uncertainty surrounding access to therapy, prognoses, and disease progression. Caregivers interviewed for this study also elucidated the time‐consuming nature of caring for a child with an LSD, particularly those who have physical and/or mental disabilities. Caring for a child with a rare disease involves long hours and flexibility, which often meant one parent could not maintain paid employment or could only work part‐time. Participants described this situation as financially draining because it inhibited the family's sources of income (Box 3, [3b]).

Expenses associated with attending frequent medical appointments and treatment sessions were described as another hidden cost that quickly accumulate and become a substantial financial burden. This was compounded by uncoordinated care, which generates additional costs for spending excessive amounts of time attending appointments, travel costs, and taking additional absences from work (Box 3, [3c]). These expenses increase exponentially for out‐of‐town travel when there are no specialist physicians or health centers in the patient's place of residence. These expenses often include gas, hotels, meals, and tickets for ferries, trains, or planes (Box 3, [3d]).

Moreover, LSD patients often require adaptations to their homes and vehicles to accommodate physical and/or mental limitations. Many patients incur additional expenses for essential healthcare and social services, such as psychotherapy, occupational therapy, and physiotherapy. Such costs and services all increase the financial strain on patients and their families (Box 3, [3e]).

Finally, participants noted that their lack of knowledge about financial support and available resources contributed to their sense of financial hardship. Most participants asserted that they failed to receive information or guidance about accessing health and social support services when they received an LSD diagnosis. This intensified the financial burden that patients and their families faced in the years following diagnosis (Box 3, [3f]).

#### 3.2.4 Theme 4: Access to social support services

Patients with LSDs and their families encounter significant hurdles in accessing the social support services they need to manage their conditions, including informational, financial, and psychological support. This is because these services are generally designed to address common diseases and lack the flexibility required to accommodate the specific needs of patients with rare diseases. This is exacerbated by the protracted timelines to receive a correct rare‐disease diagnosis, as undiagnosed patients are often denied social services (Box 4, [1a]). However, difficulties also remain after a patient has been correctly diagnosed, as the social services involved in managing a rare condition are insufficiently available or even inaccessible in some social and welfare systems.

##### Subtheme 1: Access to psychological support

Most of the participants declared that they were not offered any professional psychological support throughout their journey with the disease.

Very few participants were offered professional psychological after receiving a diagnosis (Box 4, [1b]). When such support was offered at the time of diagnosis, it was often rejected. This rejection was primarily because participants were overwhelmed by the diagnosis, unable to appreciate the importance of such support at that time, and their time was occupied by frequent medical appointments, efforts to access orphan drug therapy, and research into necessary medical interventions (Box 4, [1c]). Study participants asserted that psychological support should be offered to all patients and families at the time of diagnosis. However, this support, participants argued, should continue into the initial years following diagnosis to allow people time to adapt to demands of disease management.

Participants acknowledged the beneficial role played by patient support groups and organizations. Participants particularly noted that these entities helped patients and their families understand their diseases and adapt to the reality of living with an LSD. These patient organizations and groups often provided information about psychological support that was available to patients and their families, and they also served to connect people affected by similar conditions through a range of activities and platforms, such as support groups, conferences, and regional family days. Through these modes of communication, patients had the opportunity to speak to people with similar experiences, which often reduced their feelings of isolation and abnormality. Patient organizations also facilitated information exchange between people affected by the same condition, which helped patients and their families learn about available resources, navigate health and social systems, and manage specific diseases (Box 4, [1d]).

##### Subtheme 2: Access to tangible support

In this study, tangible support refers to the material means of financial assistance and support for patients with rare diseases that are funded by provincial programs and governments. Study participants described tangible support as one of critical elements of disease management. They articulated four specific needs for tangible support: (1) supported employment; (2) travel expenses; (3) respite services; and (4) disability support programs and services, including assistance for the families of children with disabilities, assistance for adult patients with disabilities, respite services, and adapted housing and vehicles.

In their interviews, many participants acknowledged that they had received some form of financial assistance from the government, but this varied significantly between provinces. The lack of tangible support compounded their financial, emotional, and social burdens. However, participants, especially the caregivers of children with disabilities, emphasized that benefiting from such programs was the result of extensive battles with the system to approve access to this kind of support (Box 4, [2a]).

###### Supported employment

Participants reported that supported employment, which helps patients and their caregivers gain and maintain jobs, had a major impact on LSD patients' finances. For instance, many patients and caregivers noted the importance of being eligible for sickness benefits, which constitutes one form of employment support. Indeed, most participants in this study asserted that their allocated sick leave and vacation days were rapidly exhausted by attending medical and surgical appointments, including weekly or bi‐weekly ERT infusions. In such cases, participants often felt compelled to resign from their jobs (Box 4, [2a]). This strain was often augmented by the hidden costs of managing complex diseases, such as LSDs, which are often overlooked in health policies and programs.

###### Travel expenses

Participants also reported receiving limited or no financial support to cover their travel expenses due to either a lack of available funding or participants' lack of knowledge about available resources. Moreover, participants indicated that travel expenses incurred for educational purposes (i.e., conferences, patient groups' meetings and activities) were rarely reimbursed despite being an invaluable source of information and psychological support for them (Box 4, [2b]).

###### Disability support programs and services

In this study, disability support refers to provincial programs and services that provide financial support to people with rare diseases, particularly those with physical and/or mental disabilities. Disability support programs included assistance for the families of children with disabilities, adult patients with disabilities, respite services, and adapted housing and vehicles. During discussions about the support that interviewees had received from the government to help cover the costs of these services, participants—especially parents of high‐needs children—shared an extremely long and difficult experience to accessing these services (Box 4, [2c]). They recounted fighting the system for approval to access some of the benefits of these programs due to their stringent eligibility criteria (Box 4, [2d]). Moreover, these programs only provide financial support for low‐income families (Box 4, [2e]). Essentially, middle‐income families are disadvantaged by the current system since income is a main criterion for such funding.

#### 3.2.5 Theme 5. Access to orphan drugs

A major hurdle participants encountered during their rare‐disease journey, after procurement of an accurate diagnosis, was obtaining their orphan drug, if it was available. Patients and families experienced another period of stress and anxiety after diagnosis stemming from healthcare system hindrances and drug coverage uncertainties, including whether these expensive drugs will be reimbursed by public or private insurance systems.

**FIGURE 4:**
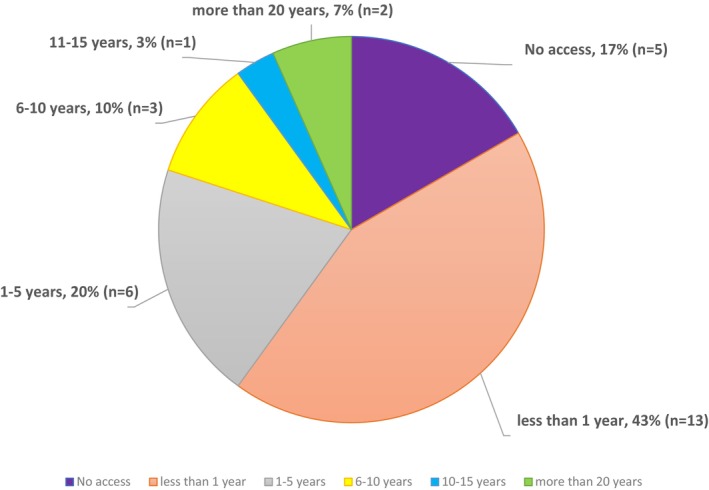
Time to access orphan drug following diagnosis. This figure illustrates the duration (in years) participants waited to access orphan drugs and commence treatment following an accurate diagnosis.

The time period between diagnosis and the start of treatment varied according to age and the type of LSD. In this study, older patients reported waiting longer to access treatment because their treatments were not available at the time of diagnosis. When treatments did become available, patients' drug applications were rejected for various reasons, but, mainly, due to a lack of evidence concerning the therapeutic and/or cost effectiveness of the new drug. Of the 30 participants in this study, 13 (43%) accessed their orphan drug within less 1 year of their diagnosis; 6 participants (20%) reported waiting 1 to 5 years for access, three participants (10%) indicated that they had waited 5 to 10 years, and one participant (3%) waited 10 years or more. Two of the 30 participants waited over 20 years for treatment to begin. Moreover, five participants (17%) reported they had not received any treatment because it was unavailable to them (i.e., MPS III) or they were denied access to it. Figure 4 illustrates the period of time participants waited to start treatment following their diagnoses.

##### Subtheme 1: The drug application process

Most participants reported that their treating physician prepared drug applications on their behalf and submitted them to relevant regulatory bodies (Box 5, [1a]). However, participants lamented the lack of a system through which they could follow up on and check the status of their drug applications (Box 5, [1b]). Furthermore, although there are currently no formal appeal processes to respond to negative decisions concerning orphan drug reimbursement, patients could appeal such decisions with the help of their treating physicians and/or patient organizations (Box 5, [1c]). At times, media exposure was utilized to increase pressure on decision‐makers and prompt them to reverse negative decisions (Box 5, [1d]).

For drug renewals, patients are generally required to undergo intensive periodic medical reassessments (usually every 6–12 months) to confirm improvements in their clinical conditions and to ensure such patients remain eligible for treatment. Notably, participants whose drugs were funded through private insurance companies experienced higher levels of insecurity, uncertainty, and stress about drug coverage continuation since continued approval for private insurance coverage depends on a combination of clinical improvement and several other factors, such as the patient or their parents maintaining their employment status and meeting the age requirements for privately insured drug coverage (Box 5, [1e]).

##### Subtheme 2: Challenges and barriers to accessing orphan drugs

Although the exorbitant cost of accessing innovative therapies for rare diseases has been already identified as the main barrier to accessing orphan drugs,^13^, ^22^, ^23^ findings from this study revealed additional challenges and barriers. These challenges, which were encountered in all provinces, include: (1) overly complex, vague, and confusing drug application process; (2) unknown and/or convoluted eligibility criteria for drug reimbursement; (3) patients’ sickness and inability to self‐advocate; (4) complex administrative procedures for ERT infusion administration and drug reimbursement strategy (particularly for privately funded orphan drugs); and (5) physicians' reluctance to submit drug applications.

###### Complex, vague, and confusing drug application process

Participants described having little or no knowledge of the application processes for accessing orphan drugs. Thus, they felt uninformed about the possibilities for moving forward with an application, how long the process would take, and when they could expect to receive a response (Box 5, [2a]). Unfortunately, many patients with LSDs suffer worsening prognoses while waiting to access treatment.

###### Unknown and/or convoluted eligibility criteria for drug reimbursement

Participants felt similarly thwarted by the confusion around eligibility criteria for drugs that had received provincial‐level approval (when these criteria were available) caused by the inconsistent and convoluted eligibility criteria for drug reimbursement. Some participants reported that to be eligible for orphan drugs, they must be significantly impacted by the disease and exhibit a substantial clinical deterioration in one or more bodily systems (Box 5, [2b]). Interestingly, one patient with Pompe living in Ontario was denied access to treatment because he was too sick to meet the eligibility criteria. This patient had respiratory failure and was ventilator‐dependent (Box 5, [2c]). Conversely, another patient with Pompe living in the same province was approved for access to treatment even though this patient had respiratory failure, was ventilator‐dependent, and was too sick and hospitalized in intensive care units for 4–5 months prior to the drug approval (Box 5, [2d]).

###### Patients' sickness and the inability to self‐advocate

Even when patients successfully overcome the challenges of navigating the drug application process, they still encounter another challenge to accessing lifesaving treatments: deteriorating health conditions. Participants expressed their hassle and frustration at simultaneously battling their diseases and the government to access their lifesaving drugs (Box 5, [2e]). Patients who were too sick and did not have a family member who helped them navigate the drug access system could not proceed with their drug application.

###### Complex administrative procedures for infusion therapy administration and drug reimbursement strategy

Some participants combined experiences of the drug‐application process with descriptions of the complex administrative procedures involved in healthcare facilities' administration of infusion therapy (e.g., hospitals, infusion clinics). For instance, a patient with Pompe disease was approved for drug reimbursement after waiting more than a year for a reimbursement decision; however, this patient was denied ERT infusion administration by the healthcare facility in which they were due to receive their drug infusion (Box 5, [2f]). Moreover, even after drug funding has been approved, orphan drug access was hindered by complex arrangements for the reimbursement of these expensive drugs to patients and families, primarily when the drug was funded through private insurance (Box 5, [2 g]).

###### Physicians' reluctance to submit a drug application

Interestingly, some participants faced challenges accessing orphan drugs that stemmed from treating physicians' reluctance to submit drug applications after patients had received correct diagnoses (Box 5, [2 h]). The reasons for the reluctance remain unclear, but some participants reported having to ask repeatedly and even beg their treating physicians to submit drug applications on their behalf (Box 5, [2i]). This was mainly the case when the drug application process and/or eligibility criteria were unclear. Notably, these patients eventually received approval for drug reimbursement when their treating physicians then submitted drug applications on their behalf.

##### Subtheme 3: Patient engagement in the orphan drug lifecycle

In this study, the term “orphan drug lifecycle” refers to the different stages of a drug cycle, which begins with pre‐clinical and clinical trial phase followed by the regulatory review and reimbursement decision‐making phase, then it enters the use in clinical practice phase, and, finally, it goes through post‐marketing studies to collect long‐term information about a drug's safety and effectiveness.^27^

This study reveals that patients and their families are keenly interested in engagement in all stages of the orphan drug lifecycle. Participants emphasized their unique ability to contribute to research, development, and evaluation of orphan drugs' drugs, including assessment of drug benefits, safety, and effectiveness in the pre‐and post‐market periods (Box 5, [3a]).

Many participants recounted actively seeking opportunities to provide insights for orphan drug regulatory review processes at both provincial and federal levels. However, most participants reported a lack of engagement in these processes. Some members of patient organizations reported their experiences of involvement with regulatory review processes, which they gained through opportunities to serve on advisory committees, by participating in or presenting webinars hosted by the government or patient organizations, and by forming patient‐group coalitions to lobby the government for orphan drug funding. Nevertheless, these involvement opportunities were informal and ad‐hoc, and their impact was unclear (Box 5, [3b]).

Participants also lamented a complete lack of patient engagement in drug reimbursement decision‐making processes, which were conducted on a case‐by‐case basis (Box 5, [3c]). Instead, participants' involvement was limited to lobbying activities, contacting government officials in person or via mail, and utilizing the media to pressure government funding for lifesaving drugs (Box 5, [3d]).

Finally, we found that participants were generally eager to engage in all research activities. Such engagement, however, was often impeded by these patients and their families not being aware of relevant opportunities and/or not being eligible to participate in relevant studies. Knowledge of the latest research activities and results was described as empowering because this allowed patients and their families to discuss potential new treatments with their doctors and thus advocate for their care. For participants who were engaged in research activities, this was mainly limited to participation in clinical trials, joining registries, and providing samples to biobanks. Very few participants had been involved in pre‐and post‐clinical trials, and when this did occur, the participant's role was limited to data collection and surveys.

## 4 DISCUSSION

The patients with LSDs and their families who participated in this study related a wide array of experiences, needs, and challenges, which were categorized into five overarching themes.

Although LSDs involve a wide variety of symptoms, complications, and prognoses, these conditions share several common aspects and challenges with other rare diseases. Likewise, experiences shared by study participants are comparable to those of individuals with other rare disorders in Canada and/or other countries. However, this study revealed some unique and underrecognized challenges and needs of people with complex rare conditions with intensive treatment regimens, such as LSDs.

### 4.1 The complexity of the diagnosis process

Our study shows how obtaining an accurate diagnosis constitutes a major challenge for patients with rare diseases. For some participants, diagnosis took as long as 40 years. Misdiagnosis was common, especially for patients with late‐onset diseases (e.g., Pompe, Fabry, and Gaucher), who sometimes received more than 20 misdiagnoses. Most patients with early‐onset diseases (e.g., MPS I, MPS II, MPS III, MPS IV, MPS VI) obtained an accurate diagnosis in less than 5 years due to developmental delays, distinctive facial features, and/or physical and growth abnormalities that triggered the diagnosis. Our findings align with studies addressing the diagnosis of rare‐disease patients in the United Kingdom (UK),^3^ Australia,^4^, ^5^ and Europe.^1^ These studies uphold that misdiagnoses and delayed diagnoses more significantly challenge patients affected by late‐onset rare diseases than those affected by early‐onset diseases.^1^, ^3^, ^4^, ^5^ Other studies also support the notion that misdiagnosis and delayed diagnosis lead not only to worse clinical conditions and outcomes but also to delayed treatment, psychological and financial damage, and reduced quality of life.^1^, ^5^, ^9^

### 4.2 Navigation of healthcare systems

The literature supports our findings related to patient and family experiences navigating healthcare systems for rare disease management. Participants reported organizational, financial, and personal barriers to healthcare services.^1^ The greatest barriers for this patient population included difficult access and referral to specialist services, prolonged waiting times, vast travel distances, and personal costs incurred by disease management.^3^, ^8^, ^9^, ^10^ Even after patients obtain an accurate diagnosis, many specialist services remain unavailable or are inadequately covered by their respective healthcare systems.^1^, ^2^, ^3^

Our findings also align with previous studies that argue the negative impact of inadequate coordination of care.^2^, ^4^, ^8^, ^9^ Lack of coordinated care contributes to poor communication between healthcare professionals and can even lead to serious mismanagement of patient care. Uncoordinated care distresses patients and their families and wastes their time and resources and those of the healthcare system.^2^, ^4^, ^8^, ^9^ Based on the participants' experiences in our study, weekly or biweekly ERT infusions lacked coordination with other medical appointments. Thus, this lifelong therapy and the costs related to traveling for it and taking absences from work became burdensome. This finding asserts the need for designated rare‐disease care coordinators and the importance of establishing rare‐disease reference centers in Canada to ensure efficient access to healthcare and sufficient coordination between numerous healthcare providers involved in patient care.

Other studies addressing patient–physician interaction present findings similar to our study regarding rare disease patients' negative experiences with non‐specialist healthcare systems and providers, whose knowledge about rare diseases and their management tends to be limited.^9^, ^10^ These limits contribute to patients' diagnostic odysseys,^1^ negatively impact patient–physician relationships, and significantly inhibit the provision of innately complex healthcare services required to manage rare diseases.^24^ Some patients described difficulty convincing their doctors that their symptoms were real and not ascribed to psychological conditions.^3^ Nevertheless, the reluctance of medical specialists to submit a drug application is not mentioned in the extant literature. This finding corroborates that a patient engagement framework is needed to meaningfully involve patients in the decision‐making for their care, including that surrounding access to lifesaving drugs.

Our findings reinforce the conclusions presented by the studies addressing access to informational support upon diagnosis.^1^, ^2^, ^3^, ^4^, ^8^ Participants in our study lamented the initial lack of direction regarding navigating health and social‐support systems, which was compounded by participants' dissatisfaction with the information they eventually received about their diagnosis.^1^, ^3^, ^4^, ^8^ Our findings confirm that receiving disease‐ and system‐related information upon diagnosis is critical for patients and their families^2^ because it facilitates optimal care and financial and psychosocial accommodations, including adjusted future plans and expectations.^10^, ^24^ Moreover, our findings stress that disease‐ and system‐related information gleaned by attending rare‐disease conferences or by communicating with others affected by similar diseases is particularly helpful.^3^, ^5^, ^8^, ^9^ These findings suggest that a myriad of educational tools (e.g., post‐diagnosis information packages, a centralized web‐based information portal for rare diseases) is needed to inform both the medical community and people with rare diseases about specific diseases, relevant resources, and patient organizations.

### 4.3 The social, psychological, and financial implications of LSDs

The patients and families in our study unanimously reported their illnesses affecting their quality of life—emotionally, socially, and economically—which is consistent with the existing research.^1^, ^2^, ^3^, ^4^, ^5^, ^6^, ^7^, ^8^, ^9^, ^10^ The commonly reported psychological implications of living with rare diseases include stress, anxiety, depression, mood disorders, grief‐like reactions, interpersonal challenges, behavioral problems, and considerable losses over the course of the lifetime.^7^ Participants also named uncertainty as a primary stressor—uncertainty about health professionals' knowledge of their rare disorders, access and potential responses to treatment, and disease progression and prognosis.^6^ Our study found that LSDs heavily burden family dynamics, particularly distressing the parents of children with physical and/or mental disabilities.^7^, ^8^, ^9^, ^10^ These parents reported significant psychosocial distress as a result of health stigma, mainly structural stigma. They noted that educational institutions insufficiently accommodated their children, which was often exacerbated by staff lacking awareness of and training for rare diseases. Interestingly, only von der Lippe et al.^6^ systematic review noted that the lack of school accommodations for rare‐disease children resulted from health stigma.

Likewise, social isolation is documented as a major challenge for rare‐disease patients and their families.^3^, ^6^, ^7^, ^8^, ^9^ Augmenting the psychosocial strain of those with rare diseases and their families is the inability to make social adjustments due to disease‐related physical limitations and treatment constraints.

Our study also highlights the financial implications of LSDs, reinforcing the findings reported by the EurordisCare3 study,^1^ the UK Strategy for Rare Diseases study,^3^ and Weber et al.^7^ review of the literature exploring experiences of IMD patients and their families. All these studies argue that rare diseases negatively impact patients' and caregivers' incomes since they are often compelled to reduce or completely relinquish professional activities. The research also notes increased travel costs due to frequent medical appointments, the cost of adaptations to patients' homes and vehicles, and the dearth of information regarding resources for financial support.^1^, ^2^, ^3^, ^8^

Despite consistent findings, no previous study sheds light on the social, psychological, and financial implications of an LSD treatment regimen (particularly the weekly or biweekly ERT infusions) on patients' and caregivers' lives. Children committed to this lifelong treatment often miss a school day every week, accounting for approximately 20% of their schooling. By extension, patients and caregivers also miss a day of work or other life activities. Though this demanding, lifelong treatment can improve a patient's health, it also impacts patients' and caregivers' relationships, careers, and sense of freedom, leading to considerable distress and losses (i.e., loss of freedom, friends, and jobs).

### 4.4 Access to social support services

Participants in our study and previous studies note that access to social services impacts every aspect of their lives. Weber et al.^7^ assert that employing a holistic approach to care, which implicitly incorporates the patient's social context, ensures effective care for patients with rare diseases. In our findings, supported by the literature, participants expressed frustration about being denied social services, often due to the lack of a diagnosis or coverage by respective social and welfare systems.^1^, ^2^, ^4^, ^7^, ^8^, ^9^ Additionally, they reported considerable but often unmet needs for professional psychological support.^1^, ^2^, ^3^, ^4^, ^5^, ^25^ Access to tangible support, including disability support programs, adaptations to houses and vehicles, respite services, reimbursement for travel expenses, and provision of medical equipment, varied across different jurisdictions and studies. In particular, the caregivers of children with disabilities in our study recounted fierce battles to obtain access to benefits and assistance from these programs. When received, this financial assistance failed to cover their disease‐related expenses.

Our findings also demonstrate the vital role of supported employment, which helps LSD patients and their caregivers gain and maintain jobs. The lack of such support intensely harms patients' finances. Although some studies have outlined common financial strains and examined “patients” or “caregivers” illness‐related inability to maintain employment,^1^, ^2^, ^3^, ^4^ further research is needed to comprehensively understand the role of supported employment for rare‐disease patients and caregivers.

### 4.5 Access to orphan drugs

Several studies assert that “patients” access to innovative rare‐disease therapies is hindered by exorbitant costs.^13^, ^22^, ^23^ In a study focusing on people with IMD living in Canada, the UK, and the United States, Khangura et al.^9^ identified additional challenges related to treatment access, including complex regulatory approval and insurance coverage. Based on patients' own words and lived experiences, we augment this list of challenges with an in‐depth exploration of the process of accessing drugs, from the medical decision to initiate treatment to the submission of drug applications to follow‐up and appeal procedures, to finally, the decision to continue administration of the drug. We found that participants from all provinces indicated several similar barriers impeding their access to orphan drugs: complex, time‐consuming drug‐review processes; vague and confusing drug‐request application, follow‐up, and appeals procedures; ambiguous and convoluted eligibility criteria for drug reimbursement; patient inability to self‐advocate due to illness; physician reluctance to submit drug applications; and organizational barriers to drug administration and reimbursement faced by patients whose drugs are funded through private insurance companies.

Most participants in our study reported limited to no involvement in the orphan drug lifecycle, especially the regulatory drug review and reimbursement decision‐making processes. Aligning with the findings of the little research addressing patient and family engagement in regulatory review and reimbursement decision‐making, participants assert their unique ability and interest to contribute to initiatives aimed at reducing evidence uncertainties surrounding the effectiveness and safety of orphan drugs. They argued they could bolster real‐world evidence and ultimately inform drug review and reimbursement decision‐making processes.^26^, ^27^, ^28^ These studies also highlight the absence of a formal framework for patient engagement in these processes in Canada.^26^, ^27^, ^28^

Finally, our findings on participants' eagerness to participate in research reinforce data presented in previous studies.^3^, ^4^, ^8^ Most patients and families involved in these studies were keen to contribute to research related to their conditions and join patient registries for those conditions where possible. They also articulated a desire to receive information about research activities and findings related to their diseases.^3^, ^4^, ^8^ Our study supports the notion that patient organizations play an important role in research since they provide participants with current research findings and alert them to clinical trial opportunities. These organizations crucially connect rare‐disease patients with research teams around the world, benefitting both patients and researchers.^25^, ^29^ Such connections increase recruitment rates and bolster research of rare diseases and corresponding orphan drugs.

## 5 STRENGTHS AND LIMITATIONS

This study's strengths are encapsulated in its extensive compilation of qualitative data, which was obtained from a significant sample size of Canadian patients and their families with varying demographics. This diversity is reflected in the age range of participants at the time of the interview (age range 25–75), their geographical locations (seven different provinces), the age of disease onset (both early‐ and late‐onset diseases), and their types of LSDs (nine different types of LSDs).

This study is also strengthened by its data collection method, which was conducted by the main researcher. Performing all interviews by a single researcher ensured the interview process and data collection were consistent. However, a single researcher coding all the interviews could lead to bias in data analysis. To mitigate this potential bias, Lincoln and Guba^20^ recommended techniques (i.e., peer debriefing, audit trail, bracketing, and reflexive diary) were used during data collection and analysis.

Several other limitations must also be noted. For instance, the applicability of the study's results is limited by its qualitative design. Thus, the experiences recounted by LSD participants in this study do not necessarily reflect those of other Canadian adults living with other rare diseases or other parents of children affected by other rare diseases. Generalizing the needs and challenges identified in this study would require quantitative follow‐up studies that validate these findings using a representative sample. Furthermore, as this study focused on a primarily Canadian‐based sample, it must be noted that people living with LSDs in other developed countries may have substantially different experiences, including different processes to access orphan drugs, varying healthcare and social systems, and disparate budget allocations for rare diseases. Further inquiry into all these differences would yield interesting and useful insights.

One notable limitation of this study pertains to the relatively small sample size of patients afflicted with LSDs across provinces in Canada. Due to stringent privacy and confidentiality regulations, we were restricted from providing detailed patient demographic information, such as age, province of residence, and gender, which is associated with participant identity, particularly in the participant information table. This precaution was imperative to safeguard the identities of the participants. Consequently, the analysis of the influence of disease type, geographical distribution, patients' age, and age at the time of diagnosis on study results was hindered. Moreover, participants were dispersed across the large geographical expanse of Canada, further complicating the assessment of regional variations. Despite these constraints, this study represents a significant advancement toward understanding the complexities of living with a rare disease, such as LSD, in the Canadian context.

Finally, we recognize that the inclusion of 10 participants affected by Pompe constitutes one‐third of the participant pool, which might potentially influence the study outcomes by either underestimating or magnifying issues specific to this particular disease type. However, it is noteworthy that participants with late‐onset LSDs, encompassing Pompe, Fabry, and Gaucher, make up 15 out of the 30 patients in the study. Given the common challenges faced by individuals with late‐onset diseases (i.e., absence of visible physical deformities, presence of invisible symptoms such as pain and fatigue, and the intricate issue of delayed diagnosis), we believe that this grouping will not significantly impact patient representation, data analysis, or study results.

## 6 CONCLUSION

The extant literature has highlighted challenges patients and families with rare diseases may encounter throughout the disease journey; however, there are some noticeable gaps in knowledge which this study sought to address. The psychological, physical, and socio‐economic factors that shape the lived experiences of Canadians with LSDs, including their disease management, access to orphan drugs, and navigation of the healthcare system, have not been systematically investigated. This study addresses this knowledge gap and enriches understanding of patients' and caregivers' experience with rare disease management and navigation of healthcare systems. Improved understanding of LSD experiences can inform health policy and ensure healthcare and social services adequately support affected patients and their families.

This study demonstrates that Canadians with chronic, progressive, degenerative rare diseases with intensive treatment regimens, such as LSDs, are vulnerable to high levels of psychological, social, and economic hardship. The management of their complex diseases requires care and support beyond having access to orphan drugs. That is, patients and their families incur significant challenges caused by multi‐faceted barriers to accessing healthcare and social support services and a convoluted process for gaining access to orphan drugs. Experiences of health stigma and discrimination when navigating healthcare and social support systems presented an evident barrier to care, which affected patients' health outcomes and quality of life.

The results of this study underscore the necessity for a holistic approach to care that integrates RD patients' social contexts. Moreover, they highlight the significance of progressing toward a Value‐Based Healthcare (VBHC) model for LSDs, a recommendation that extends from other groups of IMDs.^29^ This approach places emphasis on holistic patient care and plays a vital role in enhancing the quality of patient care and improving health outcomes, all while managing costs efficiently.^29^ Ultimately, the findings presented this study provide policymakers with valuable information about the challenges and needs of Canadians affected by complex rare diseases, such as LSDs. Policymakers can use these findings to develop the long‐awaited national strategy for rare diseases. This strategy should address all aspects of patient care, including improved diagnosis process, efficient access to coordinated healthcare and social support services, and timely access to orphan drugs. It is equally important to update existing health and social care policies and programs to address health stigma and discrimination and increase awareness of rare diseases in the medical community and society.

## AUTHOR CONTRIBUTIONS

Nahya Awada designed, collected, analyzed, and interpreted the study data relating to experiences of patients and families affected by LSDs with regards to disease management and navigation of health systems in Canada, and wrote and edited the manuscript. Martin Holcik performed the examination of the study and was a major contributor to writing the manuscript. Both authors read and approved the final manuscript.

## FUNDING INFORMATION

No dedicated funding was obtained to complete this study.

## CONFLICT OF INTEREST STATEMENT

The authors declare that they have no competing interests.

## ETHICS STATEMENT

Since human subjects were involved in this study, ethics approval was required to interview patients with LSDs and their families. Thus, an application form and all relevant study materials were submitted to the Carleton University Research Ethics Board (CU‐REB) for review and approval. CU‐REB's approval was granted in February 2019 before rare disease associations and patients being approached, and any data being collected for this study. Ethics Protocol Clearance ID: Project # 110105.

## Supporting information


**APPENDIX S1:** Certification of institutional ethics clearance.
**APPENDIX S2:** Interview questions.

**DATA S1.** Supplemental data: example quotes.

## Data Availability

The data that support the findings of this study are available from the corresponding author upon reasonable request.
