# Supplementary material for: Patient and family experiences of lysosomal storage diseases in Canada: A qualitative interview study
Source: JIMD Rep. 2023 Dec 29;65(2):63–84. doi: 10.1002/jmd2.12403 (PMC10910218; doi:10.1002/jmd2.12403)
Supplement: Supplementary file 1 — APPENDIX S1: Certification of institutional ethics clearance. APPENDIX S2: Interview questions. [file JMD2-65-63-s002.docx]

**Patient and Family Experiences of Lysosomal Storage Diseases in Canada:**

**A Qualitative Interview Study**

## Supplemental Data: Example Quotes

#### Box 1: Complexity of the Diagnosis Process—example quotes

| **Sub‐theme 1: Delayed Diagnosis** |
| --- |
| **[1a]** Participant (29)*: “I had 23 of the 24 most common misdiagnoses. By the time I was diagnosed, I had a lot of symptoms.”* (Patient, Fabry) |
| **[1b]** Participant (27)*: “Well, I saw when he was little, probably about the age of four [months], he wasn't developing like other children around me. I noticed that he had a big head. He crawled and walked late. He fell a lot. He had developed hernias, and then he had a trigger finger. So, I had a lot of questions. And, I went to my family doctor and I asked her to refer me to a pediatrician . . . And, I think she [pediatrician] knew right away when we came in the office what my son had.”* (Parent, MPS II/Hunter)  **[1c]** Participant (21)*: “I remember when I started my first job in 1980, I applied for all the standard medical benefits through the company, and I received a letter in the mail that said I had significant levels of protein in my blood. I didn't have a clue what that meant. So, I went to my doctor, I was 22 years of age, and he said, you must have had a big meal before you had the blood test. He wrote back and said: nothing to be concerned about it, he is a healthy young man. And that was the end of it. . . I know that I've had other ear, nose and throat problems later that year, in 1980. I had a balance problem, and I woke up one day and I thought I had the flu. I was off work for three months. I went to see an ear, nose, and throat specialists. I did a whole bunch of tests and his decision, or his diagnosis was that I had something called Meniere's disease.* (P21, patient, Fabry)  **[1d]** Participant (22)*: “I wasn't feeling great, and I had some pain in my stomach and they just did a basic ultrasound to rule out a stomach ulcer. And I didn't have a stomach ulcer, but then they found these spots on my spleen and my liver . . . I kept going through more and more tests and every test... So, in the end I was actually diagnosed with lymphoma cancer. That was my initial diagnosis and that was done through a pet scan. So, in order to confirm cancer, they have to do a biopsy. And then when they did my biopsy, that's when they found out that it actually wasn't cancer. It was Gaucher disease* (P22, patient, Gaucher) |
| **Sub‐theme 2: Consequences of Delayed Diagnosis** |
| **[2a]** Participant (18)*: “I have had symptoms probably for 25 years, but I was not diagnosed until a few years ago, in 2015. I was brought into the hospital with no to minimal breathing, and they put me in ICU, and they did the testing. At that point, they determined that I had Pompe.”* (Patient, Pompe) |
| **[2b]** Participant (12)*: “My biggest problem is that [after waiting for more than 40 years to obtain an accurate diagnosis] I have a lot of heart damage! … Four parts of my heart don’t work anymore, and that causes a lot of problems.”* (Patient, Fabry) |

#### Box 2: Navigation of Healthcare Systems—example quotes

| **Sub‐theme 1: Access to Healthcare Services** |
| --- |
| **[1a]** Participant (21)*: “I have a specialist for every organ in my body. I have a nephrologist, and I have a cardiologist. I have a neurologist, I have an ear, nose and throat specialist. I have a genetic specialist. I have a gastrointestinal specialist, and on and on and on it goes.”* (Patient, Fabry) |
| **[1b]** Participant (30): *“Parents are followed by over 15 different doctors and services. Once, I counted 20!”* (Parent, MPS VI/Maroteaux-Lamy) |
| **[1c]** Participant (9)*: “There are challenges to accessing healthcare professionals in our community that know anything about Hunter Syndrome for sure. . .. Our family doctor is actually new and quite young and doesn't really know anything about it. We've had these challenges in our emergency room regarding the people on call; they Google it! That is scary!”* (Parent, MPS II/Hunter)  **[1d]** Participant (23)*: “I'm fighting with the (Province name redacted) government right now to get extra dental treatment for (Child's name redacted). I have to fight with them. I've been down to my MPP's [Member of Provincial Parliament] office recently, and they're not much help to me either. It's unbelievable in this country. It is sad!”* (Parent, alpha-mannosidosis)  **[1e]** Participant (22)*: “There's no specialist [adult genetic metabolic specialist] in (City name redacted). So, I was sent to Toronto.”* (Patient, Gaucher)  **[1f]** Participant (30)*: “*We have difficulties in access to an ophthalmologist and to have an appointment for a sleep study. We do not have a pediatric [sleep] laboratory at the Atlantics . . . We are also on a waiting list to see an ophthalmology doctor in IWK [Izaak Walton Killam health center] because our only doctor in New Brunswick just closed her door. It’s been one year and a half since we’ve seen her last, and MPS VI kids should see them once a year. The waiting list in IWK is 18 months. So, if nothing is done, we will wait three years to see an ophthalmologist! So, waiting lists are long sometimes to see doctors.” (parent, MPS VI/Maroteaux-Lamy) |
| **[1g]** Participant (27): *“We stopped the speech therapy and occupational therapy when I couldn't be on the road. I had another child, and I had a sick husband. . . I couldn't be everywhere all the time.”* (Parent, MPS II/Hunter) |
| **Sub‐theme 2: Coordination of Care** |
| **[2a]** Participant (20): *“Basically, you are the main coordinator of care for your son . . . I find that definitely all the coordination of the appointments and timing of things relies a lot on me. . . I'm the one that receives the calls from the office of whatever doctor we need to meet with. And then, I have to verify that that makes sense with what I heard our geneticists say because it's such a rare disease and it has risks with anesthesia, and it's not routine to what these doctors’ offices are used to dealing with.”* (Parent, MPS IV/Morquio) |
| **[2b]** Participant (27): *“I could've been travelling like seven days a week for appointments for him [child with MPS II]. And they don't understand really that when you don't live in the city, that you can't be there every day. So, while we were at the hospital for infusion, why can't we see the pulmonologist? Why can't we meet with some of the healthcare workers while we were there for the infusion, which we’re there for five or four hours? Why can't they coordinate for us?”* (Parent, MPS II/Hunter) |
| **Sub‐theme 3: Interactions with the Medical Community** |
| **[3a]** Participant (12): *“What happened was I ended up in the hospital again with my heart. And again, they wanted to send me home ‘cause they thought I was fooling around with them.”* (Patient, Fabry) |
| **[3b]** Participant (9): *“I don't trust the medical community here to know what to do if we were in a situation that he had a disease-related complication . . . I don't know what is going to happen if we have an emergency situation cause they really don't know or understand anything about this disease and how it manifests and how it affects the different parts of him.”* (Parent, MPS II/Hunter) |
| **[3c]** Participant (20): *“Right now, it [drug application] solely lies in the hands of the geneticists. So, it puts the parents in an awkward position where you need this doctor . . . and you don't want to upset them. But, you have to go through them for any information, and they have total control. And so, it's a weird dynamic. As a parent we ultimately want him to be the best doctor for (child's name redacted) that he can be. But to put him as the chief negotiator, and he's got all the cards. He holds all the cards in this situation. And, we had to be very careful in how we got a view of those cards cause he is having phone calls and he is meeting with all sorts of people. But we had to pry the information out of him and doing that again respectfully so that we don't jeopardize our long-term relationship with him as (child's name redacted) lead doctor.”* (Parent, MPS IV/Morquio) |
| **Sub‐theme 4: Access to Disease- and System- Related Information** |
| **[4a]** Participant (01): *“We were just doing some Google work to find some information about the Morquio when we were diagnosed . . .It took time for us to understand what is going on, and what is happening, and what is the consequences, and what to do next, and what is the future. So, it took us a few years to understand all those things.”* (Parent, MPS IV/Morquio) |
| **[4b]** Participant (9): *“It takes a long time for a family to sort out how to get help with these things [social support services]. Once you're diagnosed, at diagnosis, nobody ever gives you any direction as to where to look to get some help regarding funding for travel and other necessities for your child.”* (Parent, MPS II/Hunter) |

#### Box 3: The Psychological, Social, and Financial Implications of LSDs —example quotes

| **Sub‐theme 1: The Psychological Implications of LSDs** |
| --- |
| **[1a]** Participant (10): *“It's been tough I have to say . . . It was hard not knowing what was wrong with me . . .There was like a stress level there and being limited in what you can do and [being] self-consciousness.”* (Patient, Pompe). |
| **[1b]** Participant (14): *“The most stress was coming back from the negative [reimbursement] response from the government.”* (Patient, MPS IV/Morquio). |
| **[1c]** Participant (14): *“On a daily, I would say there is kind of two stress. There is the stress you don't notice, and there's the stress that you notice. So, for example, if I have to go to a medical appointment, I'm not really stressed about the appointment itself, but how I have to get there. I have to be able to park; I have to be able to pay for parking, which is like $22 right now . . . If it's snowing, it is more stressful . . . They're kind of a background stress . . . On a daily basis, you have to manage your schedule, like medical appointments, school, work, and treatment. So, I have to manage all my activities and transport is a big concern. And also, the pain! If I'm in pain that day, that's like an added thing I have to manage.”* (Patient, MPS IV/ Morquio) |
| **[1d]** Participant (08): *“That was a big problem because I had to use my vacation to do the treatments . . . For two years, I didn't have any day off. I just worked and worked and worked. So, that was hard, that was really hard! …At the end of the second year, 2015 in December, I was not really well, and I had five days vacation left, so I went to my boss and I said I really need to take a vacation. I went to have three non-paid days that was December and he did not take it well at all! And, I had the repercussion after at my work . . . So, there were little things that I could do before that I couldn't do anymore. If we go more of a flexible time, let's say if one morning I was 15 minutes late, I stay later. . . otherwise, it goes off my vacation time. So, this is tough with a small child, and then [my work] it's far from my home, and then the traffic and the weather, so I did not have any time to relax.”* (Patient, Pompe) |
| **[2c]** Participant (29): *“The fatigue is pretty brutal. And, with other chronic pain, I had to deal with a lot of depression. So, does my disease affect my life? Well, absolutely!*” (Patient, Fabry) |
| **Sub‐theme 2: The Social Implications of LSDs** |
| **[2a]** Participant (09): *“Socially, like some of the friends that we have, we don't see anymore. In his first year of life, we were quite social with other friends that had children as well. And then he got diagnosed [at 19 months old] and I felt personally that it was almost as if those people didn't want to expose their children to a child that was going to deteriorate and die. So, we didn't see them anymore. It was almost like an immediate drop-off on being invited to any kind of outing with the same people as their children.”* (Parent, MPS II/Hunter)  **[2b]** Participant (13): *“The stature is very small. He is starting to have more and more pressure. Like he's starting to say that he realizes he's smaller than other kids . . . He’s starting to see the difference, and he talks to us about it. And, other kids at school say comments about him being smaller.”* (Parent, Pompe) |
| **[2c]** Participant (29): *“The fatigue is pretty brutal. And, with other chronic pain, I had to deal with a lot of depression. So, does my disease affect my life? Well, absolutely!*” (Patient, Fabry) |
| **[2d]** Participant (29):*“Probably the biggest impact is the amount of time I spend with healthcare professionals and/or monitoring and treating my symptoms. I would say the disease takes up a lot of my life.”* (Patient, Fabry) |
| **[2e]** Participant (16):*“So, me as a mom, socially I've lost all of social contact with friends and everything. [It’s] very busy, time consuming, preparing appointments and reports and meeting with schools, it's extremely exhausting. So, I don't really have much time for myself. . . So, yeah, it's really tough.”* (Parent, MPS I/Hurler) |
| **Sub‐theme 3: The Financial Implications of LSDs** |
| **[3a]** Participant (15): *“I lost a lot of time at work because of the symptoms that I was having. I was hospitalized many times. So yeah, I lost a lot of financial gain there because of that . . . I had to leave work for periods of time. I was often carried out at the work on an ambulance . . . I think that's the main thing, I lost a lot of time at work. One of the other reasons why it affected me financially as well [was] because I had to do this infusion every two weeks. So now, it was an 8-hour infusion. That was one day that I had to take away again off work. And so, that was a tough thing.”* (Patient, Fabry).” |
| **[3b]** Participant (05): *“The financial strains! it has been difficult to work because of the fact that when you have a child that has a rare disease, you're often seen as not a productive employee or not a consistent employee because of the fact that you're taking time off all the time for her disease and doctor's appointments. (Child's name redacted) can easily have two or three doctor's appointments every month, that is not including her enzyme replacement therapy . . .Someone needs to be there present with them for the enzyme replacement therapy. So, one [parent] is not being able to work. The other parent is the actual holder of the cost of doing the travelling.”* (Parent, MPS IV/Morquio). |
| **[3c]** Participant (03): *“[There is] at least averages about 52 trips to [hospital name redacted] per annum. . . So, ensuring that employers are able to work with that; it's about four months out of the year at work! As well as the financial cost of having to pay for weekly infusions when you were going to the hospital.”* (Parent, MPS I/ Hurler) |
| **[3d]** Participant (22): *“Every time you travelled to Toronto, that's out of your own pocket, there's no medical coverage, a private insurance plan doesn't have a section for medical travel. But, it was easy to rack up probably $2,000 in the three trips that we took. I don't drive so we would take the train. Then, we are staying at hotels. Then, you have to pay for your food when you're there.”* (Patient, Gaucher) |
| **[3e]** Participant (24): *“You're talking about vehicles and you're talking about modifications at home. There's only certain amount that the government would provide. And that was very challenging. Like a van, you need a wheelchair van, you need a lift to bring the wheelchairs into your house and all those… I mean, it's not just getting a car; not only do you have to buy a new car, but you have to pay just as much as a brand-new van to get it modified so it has a ramp so you can put your wheelchair in. Because that's not covered by the government. That's like you're looking at a good $75,000 easy*.” (Parent, MPS III/ Sanfilippo) |
| **[3f]** Participant (09): *“The first year was the toughest financially in regard to medical travel. So, traveling three hours one way once a week, having to take both parents, stay off work, gas, and kilometers on our car. I put 20,000 kilometres on my car just driving to the hospital that first year. The meals out, gas. And we didn't have any access to any kind of funding through the government for that first year. It takes a long time for a family to sort out how to get help with these things . . . It took me probably over two years after diagnosis to really discover and sort out where I could get some financial help from different government programs.”* (Parent, MPS II/Hunter) |

#### Box 4: Access to Social Support Services—example quotes

| **[1a]** Participant (09): *“Until you get an official diagnosis, you're paying for everything on your own. And, we were lucky; we had private insurance. But, until you have an actual diagnosis, which came when [child’s name redacted] was five years old, you don't get much help. And even the help that you get, you have to be proactive, and you have to go and get it yourself.”* (Parent, MPS II/Hunter) |
| --- |
| **Sub‐theme 1: Access to Psychological Support** |
| **[1b]** Participant (12): *“The only area in which I lacked support was getting psychological help. That doesn't exist for a Fabry patient! So, we had to do this all ourselves. And, if you look at healthcare, you have to look at it from a holistic approach. So, for people dealing with Fabry disease, part of the overall healthcare is psychological management.”* (Patient, Fabry) |
| **[1c]** Participant (26): *“To be honest with you, I was offered psychological support at the hospital when [child name redacted] underwent a bone marrow transplant, but I wasn't ready. So, I don't think that the parents are initially ready to go through it at the time of diagnosis or early on when you're doing a treatment, just because treatment are diagnosis are so overwhelming. And you're trying to focus on your child and his well-being, and you can't really think beyond that at that point. So, I feel maybe if those were offered after, it might have been more efficient for everyone and more beneficial for everyone*.” (Parent, MPS I/Hurler) |
| **[1d]** Participant (23): *“We have received a lot of support and help simply by sitting down and talking with other parents who are going through the same problems as we are. Generally, sitting down with them at these conferences [conducted by patient organizations] or if we're at home, we phone them, or we email or facetime . . . The advantage of the Internet really, really changed a lot for us. Before that, we were in the darkness; we really were.”* (Parent, Alpha-mannosidosis) |
| **Sub‐theme 2: Access to Tangible Support** |
| **Supported employment**  **[2a]** Participant (27): *“Well, we lost one revenue ‘cause I stayed home. I wanted to be with him. And I also was going to enzyme replacement every Monday, and then we were doing speech and occupational therapy. And this is besides hospital visits. So financially, we had one income left in my family because of the disease.”* (Parent, MPS II/ Hunter). |
| **Travel expenses**  **[2b]** Participant (14): *“[Government should be] helping to pay the fees to get there [to conferences]. It costs you to be there! It helps because when you're at conferences you meet other patients sometimes. And, if you have a rare disorder, at the beginning it was like, “Oh, well you have this disorder, you're alone in Canada, bye.” So, that's really how you got to know other people and learn about the technical stuff.”* (Patient, MPS IV/Morquio) |
| **Disability support programs and services.** |
| **[2c]** Participant (27): *“I did get that money [financial assistance], but that was a battle again! I'm very disappointed with the way the system works; you're already devastated when you see the diagnosis. And it's been a fight the whole way through to get what your child needs.”* (Parent, MPS II/Hunter)  **[2d]** Participant (30): *“Yes, we have money every month, but this was a battle to have the disability tax credit approved. We were declined three times, and then the last appeal, they approved us. But it took us one year to get this problem sorted out. This is something that needs to stop!”* (Parent, MPS VI/Maroteaux-Lamy) |
| **[2e]** Participant (x): “*A lot of support is [intended] for families with very low incomes. So, because our income isn't so low, we're not able to tap into a lot of the respite services!”* (Parent, MPS I/Hurler) |
|  |

#### Box 5: Access to Orphan Drugs —example quotes

| **Sub‐theme 1: The Drug Application Process** |
| --- |
| **[1a]** Participant (12): *“She [the doctor] did it all. She took care of everything for me.”* (Patient, Fabry)  **[1b]** Participant (02): *“There was no follow-up system at all. They [government officials] did talk to the doctor a couple of times.”* (Patient, Pompe)  **[1c]** Participant (28): *“It [the appeal] all went through the doctors.”* (Patient, Pompe)  **[1d]** Participant (27): “*And what happened is when they [government] denied it[orphan drug], the media got a hold of the story, and they help proceed with like publicizing it and trying to get it out there that the only treatment option for my son at the time was the ERT [enzyme replacement therapy].* (Parent, MPS II/Hunter) |
| **[1e]** Participant (13): *“We have the private insurance, but if we lose the private insurance, is the government gonna continue paying? I don't know because it's not clear. Nobody really knows! So, it's very stressful not to have the policy clear.”* (Parent, Pompe) |
| **Sub‐theme 2: Challenges and Barriers to Accessing Orphan Drugs** |
| **[2a]** Participant (13): *“I often feel like I'm totally from the outside. I don't really know what's going on. And then a lot of things are unclear. Even on the side of the doctors in the hospital, it's not clear for them. They're not certain! So, it seems that there's nobody specifically in charge. It's just little pieces, a little bit there, a little bit there, a little bit there.”* (Parent, Pompe) |
| **[2b]** Participant (29): *“There's basically five things you have to have. You have just had a stroke. Your pain must be intractable. And certainly, even I had a lot of pain, I hadn't tried everything. You have to have bowel disease it's so severe that it’s life-altering. I had irritable bowel disease, I mean I consider my bowel disease problematic, but it didn't hit that threshold. You have to have cardiac disease to the point that you have ventricular hypertrophy . . . And, end stage renal disease or a drop in renal function, which is how I got on it. So, my sister went on enzyme replacement immediately even though her symptoms were not as severe, but her heart disease . . . she hit the threshold for ventricular hypertrophy on echocardiogram. So, she got enzyme replacement right away. I didn't get it for three years. I had to wait till I had a drop in kidney function and then I was eligible for it.”* (Patient, Fabry) |
| **[2c]** Participant (11): “*They [government] do not fund the drug for Pompe patients on ventilator. And I am on a ventilator. So, I'm still on compassionate use through the [drug company name redacted]. The government won't approve me on the ventilator, but it [ERT] improved my quality of life on ventilator.”* (Patient, Pompe) |
| **[2d]** Participant (6): “*Well, they were trying to get me off [ventilator], I wasn't strong enough. So, I kept going into almost a coma; they couldn't wake me up. So, that happens sometimes. And one time I almost died and then they finally decided I should be ventilated at night all the time. Cause they were trying to wean me off, and it was too much. So, I was totally out. I was hallucinating all the time and kept going into almost a coma. My blood pressure would drop to almost nothing. They have to revive me! And finally, I would beg them not to do that . . . So, then the medicine, I finally got approved!”* (Patient, Pompe) |
| **[2e]** Participant (18): *“And you gotta remember, the person that has the disease is usually very sick, and they're not capable of making decisions at the time, not knowledgeable about the disease whatsoever. So, you need a spokesperson. You need someone that can be your advocate.”* (Patient, Pompe) |
| **[2f]** Participant (11): “*It took a couple of weeks to process the paper, and then you waited for a couple of months, and then they say no for the reimbursement, and then you resubmit things, and then they say yes after another couple of months. And then, when you got the drug approved for funding, the hospital said no! And, then the doctor called you, and that was like almost a year now. He called you saying that we cannot give you the medication in this hospital. And then he referred you to another doctor at the [name of university redacted], and it took you one year to process your drug infusion at that hospital, not the funding because the funding was already approved.”* (Patient, Pompe) |
| **[2g]** Participant (16): “*Our fight [was] with the reimbursement because the way the private insurance worked was that we had to prepay the hospital, so the hospital would bring it [ERT] in. Every Thursday, (child's name redacted) would go to the hospital and do ERT . . . I had to go down and pay their pharmacy before the medication was brought up where it was intravenous into his body. The problem is that these medications are very expensive! We ended up maxing out our credit limit because the insurance would take eight weeks to pay them back. So it was probably about $3000 at the time. So, 3000 times eight, you could imagine how much money was on the credit card. So, our struggle was more with that part of it . . . I had to call all my credit card companies and max up my limits to accommodate the wait time. And we got all our money back; It's just the gap in between. Not Everybody has $3,000 in a week. So that was a big issue!”* (Parent, MPS I/Hurler) |
| **[2h]** Participant (18): “*I think the biggest issue for me is that the doctor, the specialist (name redacted) I was seeing, tried to deter me from getting the treatment. He told me twice that it would not be of any benefit to me. It wasn't until I went away to a conference that I realized that all these people were taking the treatment. So, I came back and then we went further with it through the private company.”* (Patient, Pompe)  **[2i]** Participant (22): “*I told the doctor I wanted to take it [the orphan drug], and like he said no, and then I started to beg, and he said okay. I feel like there's a lot of push to do. So, we're sort of left to the doctors.”* (Patient, Gaucher) |
| **Sub‐theme 3: Patient Engagement in Orphan Drug Lifecycle** |
| **[3a]** Participant (14): *“I think insights provided by patients are very valuable. The regulators and the decision-makers don't know the specific things that really concern the patient.”* (Patient, MPS IV/Morquio) |
| **[3b]** Participant (4): *“I work with the Canadian Organization of Rare Disorders, and we've lobbied the government on behalf of many rare disease patients. The opinion of that organization and myself is that we shouldn't have to fight individually for approval. It should be approved on a disease-by-disease basis. So, once you approve Myozyme for me, you approve Myozyme for all patients who meet the criteria, and there are criteria to meet. . .. It [meeting with government officials] is not a formal process. They were ad-hoc committees based on patient input to criteria for approval of the drug. They're asking what we, as patients, would accept as criteria to approve the use of the drug . . . We met with the Ontario government to work out what . . . [those] criteria [were]. And, the input was not just from patients’ input; it was from medical staff, experts in the field, the drug company.”* (Patient, Pompe)  **[3c]** Participant (20): *“We were not invited in; it is just the doctors . . .. Families are left out.”* (Parent, MPS IV/Morquio) |
| **[3d]** Participant (28): *“Myozyme was approved through the CDR [Common Drug Review conducted by CADTH]. I’d gone to my local MLA [Member of the Legislative Assembly], who blew me away. He's also a physician here in our town, and he basically blew me off because it was too expensive. And, even given him proof and giving him the articles that were published in the US, he basically said, anybody can come up with these kinds of numbers and figures and is it true?” . . . I tried numerous times to get into see the Minister of Health in our province, but they refused to see me and would send me off to speak to somebody in [province name redacted] drug program, who basically said the same thing: There's no proof that it works. Even though it's funded in numerous countries, there's no proof that it works! It's like, well, what more proof do you want? Well, even if it does get approved by the CDR, there is still no!”* (Patient, Pompe) |
