# Supplementary material for: Patient and family experiences of lysosomal storage diseases in Canada: A qualitative interview study
Source: JIMD Rep. 2023 Dec 29;65(2):63–84. doi: 10.1002/jmd2.12403 (PMC10910218; doi:10.1002/jmd2.12403)
Supplement: Supplementary file 2 — DATA S1. Supplemental data: example quotes. [file JMD2-65-63-s001.docx]

## Supplemental Data: Appendices

### Appendix 1: Certification of Institutional Ethics Clearance

### Appendix 2: Interview Questions
